# Rolling Based on Multi-Source Time–Frequency Feature Fusion with a Wavelet-Convolution, Channel-Attention-Residual Network-Bearing Fault Diagnosis Method

**DOI:** 10.3390/s25134091

**Published:** 2025-06-30

**Authors:** Tongshuhao Feng, Zhuoran Wang, Lipeng Qiu, Hongkun Li, Zhen Wang

**Affiliations:** 1 School of Mechanical Engineering, Dalian University, Dalian 116622, China; fengtongshuhao@s.dlu.edu.cn (T.F.); wangzhen@dlu.edu.cn (Z.W.); 2 School of Instrument Science and Engineering, Southeast University, Nanjing 210096, China; 213232086@seu.edu.cn; 3 School of Mechanical Engineering, Dalian University of Technology, Dalian 116024, China; lihk@dlut.edu.cn

**Keywords:** rolling bearing, fault diagnosis, time–frequency diagram, wavelet convolution channel attention residual network, attention

## Abstract

As a core component of rotating machinery, the condition of rolling bearings is directly related to the reliability and safety of equipment operation; therefore, the accurate and reliable monitoring of bearing operating status is critical. However, when dealing with non-stationary and noisy vibration signals, traditional fault diagnosis methods are often constrained by limited feature characterization from single time–frequency analysis and inadequate feature extraction capabilities. To address this issue, this study proposes a lightweight fault diagnosis model (WaveCAResNet) enhanced with multi-source time–frequency features. By fusing complementary time–frequency features derived from continuous wavelet transform, short-time Fourier transform, Hilbert–Huang transform, and Wigner–Ville distribution, the capability to characterize complex fault patterns is significantly improved. Meanwhile, an efficient and lightweight deep learning model (WaveCAResNet) is constructed based on residual networks by integrating multi-scale analysis via a wavelet convolutional layer (WTConv) with the dynamic feature optimization properties of channel-attention-weighted residuals (CAWRs) and the efficient temporal modeling capabilities of weighted residual efficient multi-scale attention (WREMA). Experimental validation indicates that the proposed method achieves higher diagnostic accuracy and robustness than existing mainstream models on typical bearing datasets, and the classification performance of the newly proposed model exceeds that of state-of-the-art bearing fault diagnostic models on the same dataset, even under noisy conditions.

## 1. Introduction

Rolling bearings, as core components of rotating machinery, are widely employed in key sectors such as aviation, energy, and manufacturing, with their condition directly influencing the reliability and safety of equipment operation [1]. According to statistics, bearing failures account for 45–55% of mechanical failures, potentially resulting in major safety incidents and significant economic losses [2]. For instance, the failure of aviation engine bearings has led to aircraft crashes, while production interruptions due to industrial equipment downtime can incur costs running into millions of dollars. Once bearings or other key components fail, the safety of the entire mechanical system is jeopardized, potentially leading to considerable economic losses or safety incidents. Therefore, investigating fault diagnosis techniques for critical components such as rolling bearings is essential for enhancing system reliability and safety.

In industrial settings, bearings’ fault characteristics are often obscured by complex vibration signals. Consequently, extracting effective fault features from non-smooth, multi-noise signals has emerged as a challenging and critical research topic.

Traditional methods primarily rely on time–frequency analysis techniques to extract fault features from signals. The short-time Fourier transform (*STFT*) achieves time–frequency localization using a fixed window; however, its resolution is constrained by the uncertainty principle, making it difficult to simultaneously capture high-frequency transients and low-frequency modulation components [3]. The wavelet transform (WT) enhances local feature extraction through multi-scale decomposition, yet its basis function selection depends on prior knowledge and displays limited adaptability to complex modulated signals [4]. To address the dependence on predetermined basis functions, the Hilbert–Huang transform (*HHT*) was introduced; it adaptively generates intrinsic modal functions (*IMFs*) via empirical mode decomposition (EMD) and integrates Hilbert spectral analysis to estimate instantaneous frequencies [5]. However, EMD is prone to modal aliasing in the presence of strong noise or similar frequency components, leading to ambiguous physical interpretations [6]. The Wigner–Ville distribution (*WVD*), a quadratic time–frequency analysis method, has attracted attention for its high resolution. *WVD* can accurately characterize the instantaneous energy distribution of a signal, making it especially suitable for detecting shock components [7]. Nevertheless, its inherent cross-term interference is particularly severe when analyzing multi-component signals, which significantly reduces the interpretability of the resulting time–frequency plots [8].

In rolling bearing fault diagnosis, both the sample size and the diversity of fault types influence not only the effectiveness of feature extraction but also the training and prediction performance of the model. Bearing failures are typically classified into various categories, such as outer ring failures, inner ring failures, and rolling element failures. The causes of these failures include insufficient lubrication, overload, and material fatigue, among other factors. Although traditional time–frequency analysis methods can extract some fault characteristics when processing complex signals, they often fall short in fully capturing the nuances of different fault types amid diverse and complex samples. Consequently, as the number of fault samples increases and fault types become more varied, traditional single-analysis methods gradually exhibit more limitations. In response, an increasing number of researchers are exploring deep learning techniques for bearing fault diagnosis with the aim of counteracting the shortcomings of traditional methods through end-to-end feature learning, thereby enhancing the overall accuracy and reliability of fault diagnosis.

In recent years, deep learning (DL) techniques have significantly improved diagnostic performance through end-to-end feature learning. Convolutional neural networks (CNNs) excel at extracting spatial features from vibration signals, long short-term memory networks (LSTMs) effectively model temporal dependencies, and Transformers are adept at managing long sequences due to their self-attention mechanism [9,10,11]. Li et al. were the first to apply neural networks for bearing vibration analysis in both the time and frequency domains [12]. Ruan et al. refined the parameter settings of traditional CNNs to boost their performance in recognizing fault features [13]. Chen et al. developed an automatic feature-learning neural network that combines CNNs with various convolutional kernel sizes to extract features from frequency signals and uses LSTM to classify bearing fault types [14]. Gao et al. employed a multichannel continuous wavelet transform to convert the original signal into multi-channel features more efficiently, thereby reducing network parameter requirements, and further utilized a convolutional feature-based RNN combined with residual and LSTM blocks to simultaneously mine temporal aspects and local vibration features, addressing the problem of weak fault signatures caused by strong noise in early faulty bearing signals [15]. However, the frequency domain resolution of conventional convolutional kernels remains insufficient, causing high-frequency shock components to be easily overwhelmed by noise [16]. To tackle this issue, researchers have introduced attention mechanisms to enhance feature representation. For example, SENet emphasizes critical frequency bands through channel weighting [17], while CBAM integrates both spatial and channel attention [18]. Ding et al. proposed a novel time–frequency Transformer model to optimize the representation of vibration signals within a comprehensive end-to-end bearing fault diagnosis framework, thereby overcoming the limitations of convolutional and cyclic structures in terms of computational efficiency and feature representation [19]. Jin et al. introduced a time series Transformer-based method that mitigates the long-term dependency issues of traditional CNNs and RNNs in fault diagnosis, further improving bearing fault identification capability [20]. Wang et al. combined the channel attention mechanism of squeeze excitation networks with CNNs to devise a bearing fault diagnosis method based on symmetric point mapping and squeeze excitation convolutional neural networks (SE-CNNs) [21]. Lin et al. integrated CNN and Transformer augmentation to construct an end-to-end bearing fault diagnosis framework, enhancing both local and temporal feature extraction while incorporating a cross-fertilization Transformer to improve the correlation between acoustic and vibration features for more accurate identification and classification of bearing fault types [22].

Despite significant progress in time–frequency analysis and deep learning methods for rotating machinery fault diagnosis, existing research still confronts multifaceted challenges. At the signal processing level, most approaches rely on a single time–frequency transform (e.g., *STFT* or *CWT*) to generate input features, which inherently limits time–frequency characterization. At the layer level, current convolutional layers suffer from limited receptive field expansion, performance saturation, and an excessive number of parameters that hinder effective local interactive learning [23]. At the model architecture level, although mainstream residual networks mitigate the gradient vanishing problem through skip connections, their stacked design introduces numerous redundant parameters, and fixed residual paths cannot dynamically adjust the contribution of cross-layer features, leading to inefficient feature fusion [24]. Existing lightweight methods reduce the number of model parameters by employing pruning or quantization; however, these techniques may compromise the model’s expressive ability and degrade accuracy [25]. Furthermore, some attention mechanisms aggregate learned weights by simple averaging, which is not conducive to enhancing the discriminative power of deep features [26].

Therefore, this study proposes a multi-source time–frequency feature-enhanced fault diagnosis method, termed WaveCAResNet (Wavelet Channel-Attention Residual Network). The method is based on the deep fusion of wavelet multi-scale convolution and a dynamically weighted residual architecture, aiming to address core challenges such as limited time–frequency characterization from single transforms, redundant network parameters, and the imbalance between model lightweight and diagnostic accuracy. In this approach, time–frequency maps generated by the continuous wavelet transform (*CWT*), short-time Fourier transform (*STFT*), Hilbert–Huang transform (*HHT*), and Wigner–Ville distribution (*WVD*) are first fused to synthesize the complementary advantages of different techniques. Inspired by the ResNet-18 architecture, a lightweight network structure—WaveCAResNet—is then designed for fault diagnosis, ensuring high diagnostic accuracy with reduced model size and parameter count, as fully validated by experiments.

The main contributions of this study are the following:

(A) Four types of time–frequency maps—namely, continuous wavelet transform (*CWT*), short-time Fourier transform (*STFT*), Hilbert–Huang transform (*HHT*), and Wigner–Ville distribution (*WVD*)—are fused and used as input features. This integration fully leverages the global stability of *STFT*, the multi-scale characteristics of CWT, the adaptive decomposition capability of HHT, and the high-resolution advantage of WVD, thereby enhancing the fault feature detection rate through feature complementarity.
(B) WaveCAResNet, a lightweight network, was designed with the introduction of the wavelet convolution layer (WTConv), as well as the channel-attention-weighted residual (CAWR), and based on EMA, weighted residual efficient multi-scale attention (WREMA) was designed. WaveCAResNet effectively integrates wavelet convolution, CAWR, and WREMA, and constructs a lightweight, efficient bearing fault diagnosis network, which effectively solves the deficiencies of the above methods.

## 2. Proposed Method for Fault Diagnosis in Bearings

**Figure 1:**
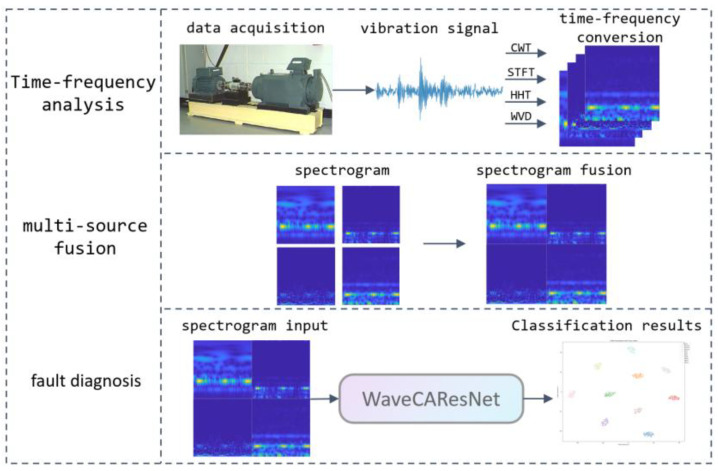
Flow of the proposed methodology.

We propose an end-to-end deep learning framework for rolling bearing fault diagnosis, as depicted in Figure 1. The framework comprises four integral components: data acquisition, time–frequency analysis, multi-source fusion, and WaveCAResNet-based fault diagnosis. In essence, it is divided into three main parts: a time–frequency analysis module, an image fusion component, and a fault diagnosis unit, each playing a crucial role in the overall process. First, the data acquisition module collects vibration signals from rolling bearings. Next, four time–frequency analysis techniques—continuous wavelet transform (*CWT*), short-time Fourier transform (*STFT*), Hilbert–Huang transform (*HHT*), and Wigner–Ville distribution (*WVD*)—are sequentially applied to convert the one-dimensional vibration data into two-dimensional time–frequency representations, such as instantaneous frequency diagrams. This transformation effectively extracts the inherent time–frequency features of the signals. Subsequently, a multi-source image fusion component integrates the resulting time–frequency images to form a comprehensive representation, thereby facilitating subsequent feature extraction and fault discrimination. Finally, the fused images are fed into a lightweight network, WaveCAResNet, which extracts image features via deep learning and outputs the fault classification results. Overall, this framework is designed to efficiently and accurately identify the operating state of rolling bearings, providing a critical basis for equipment condition monitoring.

This study proposes a fault diagnosis method for local faults in the inner ring, outer ring, and rolling elements, as well as pitting, spalling, and crack faults caused by surface damage. This method significantly improves the accuracy, precision, and recall rate of fault diagnosis and has been verified using two publicly available benchmark datasets. The key parameters of the experimental setup are shown in Table 1, ensuring the reproducibility of the experiment.

### 2.1. Data Pre-Processing

Rotating machinery fault vibration signals exhibit typical non-stationary characteristics, with their frequency components dynamically varying over time. Traditional time-domain analysis methods are inadequate for capturing the local transient features of these signals, while frequency-domain techniques lose temporal information and therefore cannot pinpoint when faults occur. In contrast, time–frequency analysis captures the dynamic evolution of the signal across both the time and frequency dimensions, providing a more discriminative two-dimensional feature representation for deep networks. However, relying on a single time–frequency method is constrained by inherent algorithmic limitations such as resolution trade-offs and cross-term interference. To overcome these shortcomings, this study proposes a multi-temporal–frequency graph fusion strategy that comprehensively leverages complementary features to enhance diagnostic performance.

To overcome the limitations of individual methods and fully exploit the complementary strengths of various time–frequency techniques, this study employs four classical time–frequency analysis methods to characterize the signal from multiple perspectives. The principles and implementation details of these methods are described as follows:

Continuous Wavelet Transform (*CWT*): The local time–frequency analysis of the signal is realized by the expansion and translation of the mother wavelet, and the expression is

(1) $$\;CWT\left(a,b\right)=\frac{1}{\sqrt{a}}\int_{-\infty }^{\infty }x\left(t\right)\psi^{*}\left(\frac{t-b}{a}\right)dt$$

where a is the scale factor (inversely proportional to frequency), b is the translation factor, and $\psi \left(t\right)$ is the mother wavelet function.

The Morlet wavelet basis function is used, and its expression is

(2) $$\psi \left(t\right)=\frac{1}{\sqrt{2\pi }}e^{i\omega_{0}t}e^{-\frac{t^{2}}{2}}$$

where $\omega_{0}$ is the center frequency and t is the time variable.

Short-Time Fourier Transform (*STFT*): The short-time power spectrum is calculated by Fourier-transforming the localized segments of the signal by adding a window, and the expression is

(3) $$STFT\left(t,f\right)={\left|\int_{-\infty }^{\infty }x\left(\tau \right)w\left(\tau -t\right)e^{-i2\pi f\tau }d\tau \right|}^{2}$$

where $w\left(t\right)$ is the window function. A Hamming window is used with a window length of 64 points and an overlap rate of 50% to balance spectral leakage with computational efficiency.

Hilbert–Huang transform (*HHT*): the signal is adaptively decomposed into a finite number of intrinsic modal functions (*IMFs*) by empirical modal decomposition (EMD), and then the time–frequency spectrum is obtained by performing the Hilbert transform on each IMF; the expression is

Empirical Modal Decomposition (EMD): The signal *x*(*t*) is decomposed into the sum of a number of intrinsic modal functions (*IMFs*):

(4) $$x\left(t\right)=\sum_{k=1}^{K}IMF_{k}\left(t\right)+r\left(t\right)$$

Hilbert spectrum calculation:

(5) $$H\left(f,t\right)=\sum_{k=1}^{K}IMF_{k}\left(t\right)\cdot e^{i\int f_{k}\left(t\right)dt}$$

where $IMF_{k}\left(t\right)$ represents the kth IMF component, which satisfies the local symmetry and the limit on the number of extreme points. $r\left(t\right)$ is the residual component, which characterizes the signal trend term, and $f_{k}\left(t\right)$ represents the instantaneous frequency of the kth IMF component, computed by the Hilbert transform.

Wigner–Ville Distribution (*WVD*): *WVD* is a bilinear time–frequency analysis method, which calculates the instantaneous energy distribution through the autocorrelation function of the signal, and the expression is

(6) $$WVD\left(t,f\right)=\int_{-\infty }^{\infty }x\left(t+\frac{\tau }{2}\right)x^{*}\left(t-\frac{\tau }{2}\right)e^{-i2\pi f\tau }d\tau$$

where $x^{*}()$ represents the complex conjugate of the signal, which is used to construct the resolved signal, and τ represents the time delay variable, which indicates the autocorrelation time offset of the signal.

Continuous Wavelet Transform (*CWT*) achieves adaptive time–frequency resolution adjustment through the expansion and translation of wavelet bases, making it particularly effective in detecting transient shocks. However, its frequency resolution in the low-frequency region is limited and depends on the a priori selection of wavelet bases. In contrast, the Short-Time Fourier Transform (*STFT*) offers efficient steady-state spectral analysis using a fixed window function, yet it struggles to adequately capture the time–frequency resolution of rapidly changing non-stationary signals. The Hilbert–Huang transform (*HHT*), which is based on adaptive EMD decomposition and Hilbert spectral analysis, can process nonlinear signals without predefining basis functions, but it is computationally intensive, sensitive to noise, and prone to interference from modal aliasing. Meanwhile, the Wigner–Ville Distribution (*WVD*) maintains high instantaneous frequency resolution and adapts well to the analysis of multicomponent signals, although it still suffers from slight time–frequency blurring and requires careful parameter tuning.

To enable the complementary integration of multimodal time–frequency features, this study proposes a multi-source time–frequency map fusion strategy, where four time–frequency maps are arranged in a 2 × 2 grid. The specific process is outlined as follows:

First, the dimensions were standardized. The time–frequency maps generated by *CWT*, *WVD*, *HHT*, and *STFT* were scaled to a uniform size of 224 × 224 pixels, ensuring spatial consistency.

**Figure 2:**
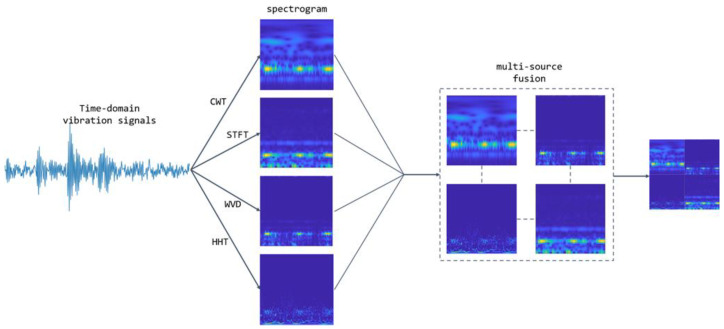
Data preprocessing process.

During the spatial fusion process, as illustrated in Figure 2, a synthetic map is constructed. In the top row, the *CWT* (left) and *WVD* (right) are aligned horizontally to capture the correlation between transient shocks and transient frequencies, while in the bottom row, the *WVD* (left) and *STFT* (right) are aligned horizontally to jointly suppress cross-terms and retain the steady-state spectral baseline. The final composite image, with dimensions of 224 × 224 pixels, is generated and converted into an RGB three-channel input for the network.

### 2.2. A Lightweight Model WaveCAResNet

In this study, we propose a novel neural network model called WaveCAResNet, which is based on wavelet convolution and a weighted residual attention mechanism. The model aims to improve the accuracy and robustness of bearing fault diagnosis by integrating time–frequency feature extraction, dynamic channel attention, and temporal feature enhancement strategies. WaveCAResNet incorporates three key components: the wavelet convolutional layer (WTConv) for multi-frequency domain feature extraction, the channel-attention-weighted residual mechanism (CAWR) for cross-channel feature optimization, and the improved weighted residual efficient multi-scale attention module (WREMA) for temporal feature fusion. This integrated approach enables the efficient modeling of complex vibration signals. Compared with traditional convolutional neural networks, WaveCAResNet offers several significant advantages: (1) It enhances the model’s adaptability to non-smooth signals by jointly extracting time–frequency domain features through the wavelet convolutional layers; (2) it suppresses noise interference by dynamically focusing on key feature channels via the CAWR module; (3) it captures long-term dependencies in time-series data by fusing historical and current features using the WREMA module.

#### 2.2.1. Wavelet Convolution Layer

**Figure 3:**
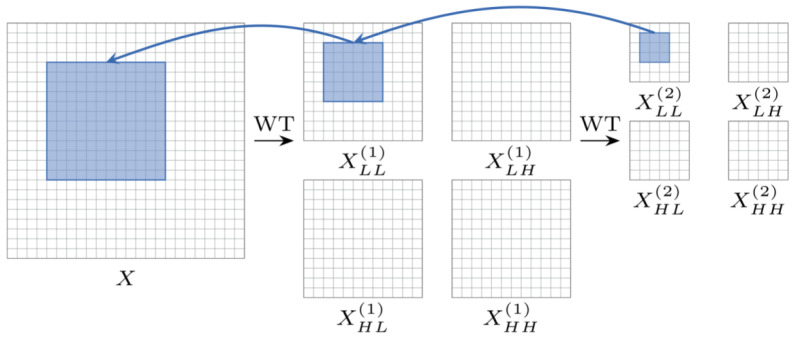
Performing convolution in the wavelet domain leads to a larger receptive field for multi-scale time–frequency feature extraction, where $X_{LL}$ is the low-frequency component of the input image *X*, $X_{LH}$ is the high-frequency component of the input horizontal direction, $X_{HL}$ is the high-frequency component of the input vertical direction, and $X_{HH}$ is the high-frequency component of the input diagonal direction. Among them, (1) represents the first-level wavelet domain, and (2) represents the second-level wavelet domain.

Conventional convolutional operations have limited-sense field expansion and tend to ignore the frequency domain characteristics of the signal when extracting features in the time domain. For this reason, this study introduces a wavelet convolution layer (WTConv) based on the method proposed in [27]. As shown in Figure 3, the wavelet convolution layer (WTConv) achieves multi-scale time–frequency feature extraction by combining the continuous wavelet transform (*CWT*) with convolutional operations, which permits the use of smaller convolutional kernels to operate over a larger region of the original input. Specifically, the input signal is subjected to multi-stage wavelet decomposition to generate low- and high-frequency sub-bands, each of which is feature-mapped by a separate lightweight convolution kernel, and finally, the features are reconstructed by the inverse wavelet transform. The process can be expressed as

(7) $$\;Y=IWT\left(\sum_{i=1}^{l}rConv\left(W_{i},WT_{i}\left(X\right)\right)\right)$$

where $WT_{i}$ denotes the *i*-th level of wavelet decomposition and $W_{i}$ is the parameter of the corresponding convolution kernel. WTConv uses a cascade decomposition strategy such that the receptive field of the *k* × *k* convolution kernel grows exponentially with the decomposition level *ℓ*($2^{l}\cdot k$), while the number of parameters only increases linearly ($l\cdot k^{2}\cdot c$). Experiments show that this method significantly improves the model’s ability to capture low-frequency features while preserving local details.

#### 2.2.2. Channel-Attention-Weighted Residual (CAWR)

**Figure 4:**
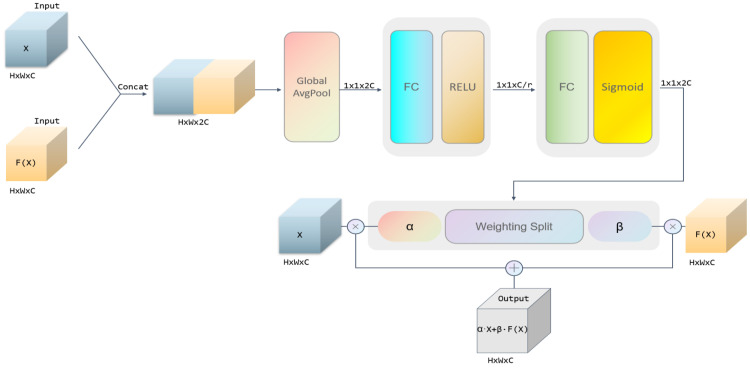
Schematic diagram of CAWR.

To enhance the model’s focus on key feature channels, we propose the Channel-Adaptive Weighted Residual (CAWR) module. This module generates channel weight coefficients using global average pooling and nonlinear mapping and dynamically adjusts the fusion ratio between the original and residual features. As shown in Figure 4, the computational flow of the module can be summarized in the following steps: First, the input features and the residual features are concatenated along the channel dimension to form joint features. Next, global average pooling compresses the spatial information and extracts global statistics at the channel level. Then, a fully connected layer combined with a nonlinear activation function generates two sets of dynamic weight coefficients, corresponding to the channel importance of the original and residual features, respectively. Finally, these weight coefficients are applied in a channel-by-channel weighted sum of the two types of features to produce the fused output features.

Specifically, the module takes two inputs: the original feature x and the residual feature $f_{x}$. These features are concatenated along the channel dimension to form the joint feature $X_{concat}$, which aligns both feature types and serves as a foundation for subsequent global information modeling. Next, global average pooling is applied to the joint features to compress the spatial dimension by averaging the values at all spatial locations for each channel, resulting in the channel description vector z, which is mathematically expressed as

(8) $$\;z=\frac{1}{H\times W}\sum_{i=1}^{H}\sum_{j=1}^{W}X_{\mathrm{concat}}\left[:,:,i,j\right]$$

This step transforms complex spatial information into compact channel statistics that provide global contextual information for weight generation.

Dynamic weight generation is the core component of the module. The channel description vector *z* is processed through two fully connected layers, accompanied by activation functions to generate normalized attention weights. Initially, the feature dimensions are reduced via a fully connected layer, with the nonlinearity introduced by a ReLU activation function. Next, the dimensions are restored using another fully connected layer, and the resulting weight values are constrained to the range [0, 1] by applying a Sigmoid function. The final generated weight vector, w, can be expressed as

(9) $$\;w=Sigmoid\left(W_{2}\cdot ReLU\left(W_{1}\cdot z\right)\right)$$

where $W_{1}$, $W_{2}$ are the trainable parameters of the fully connected layer inside the module, whose role is to dynamically generate the fusion weights of original and residual features by learning the channel dependencies in the data.

The weight vector w is split into two independent sets of coefficients and beta, which correspond to the channel-wise importance of the original feature x and the residual feature $f_{x}$, respectively. The split weights are extended to the spatial dimensions of the original features through the broadcast mechanism and are subjected to Channel-wise Multiplication with the corresponding features. The final output features are obtained by summing the weighted two-part features:

(10) $$Output=\alpha \cdot x+\beta \cdot f_{x}$$

**Figure 5:**
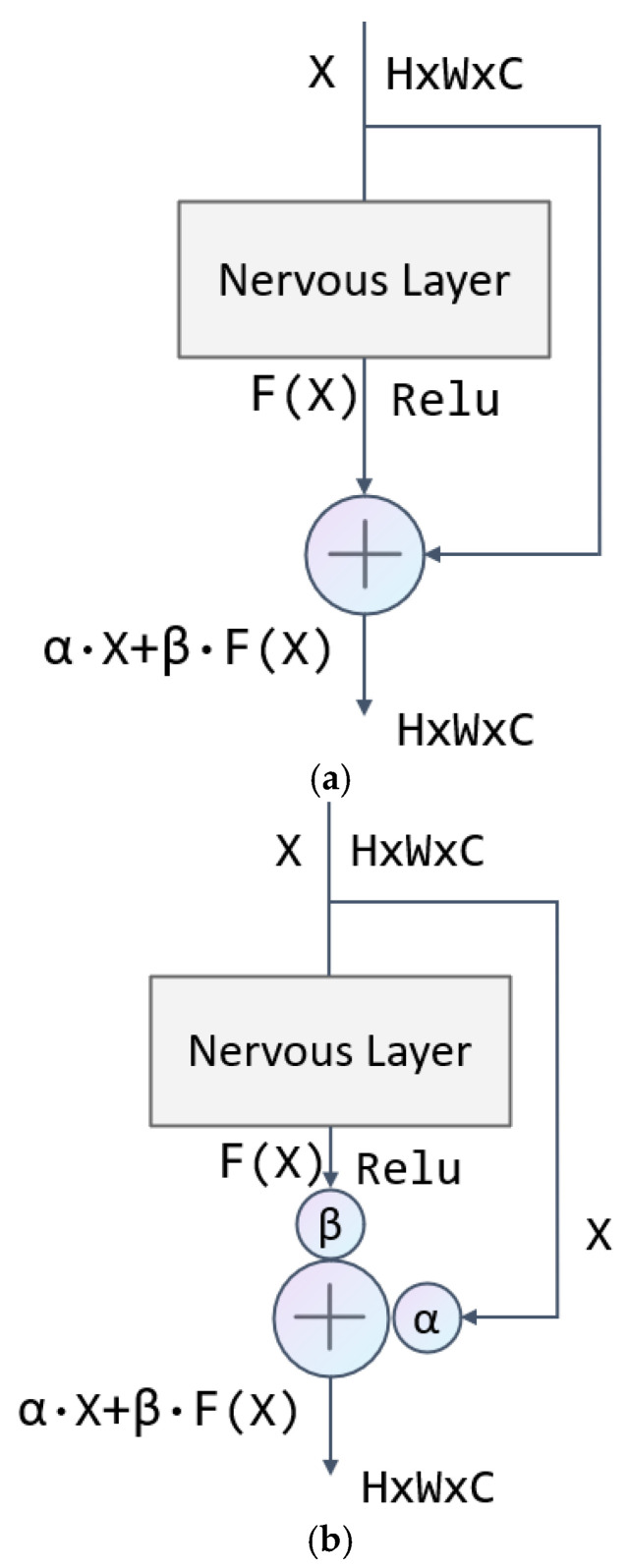
Residual structure: (**a**) original residual structure and (**b**) weighted residual structure using CAWR mechanism.

By adding two learnable parameters to the traditional residual structure, dynamic feature fusion is realized, as shown in Figure 5. By introducing the CAWR mechanism, it solves the shortcomings of the traditional residual structure due to its simple addition operation only in each jump connection, lacks the ability to model the complex relationship between features and enhances the model’s ability to model the relationship between features.

#### 2.2.3. Weighted Residual Efficient Multi-Scale Attention (WREMA)

**Figure 6:**
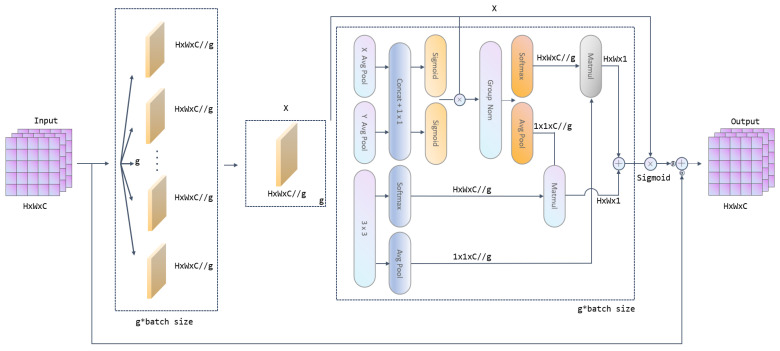
Schematic diagram of WREMA.

In order to enhance the model’s ability to model the time-series dynamic characteristics of vibration signals, Weighted Residual Exponential Moving Average (WREMA) is proposed based on [28], aiming to improve the model’s ability to model the time-series dependencies in bearing vibration signals by dynamically fusing historical features with current features. The core process is divided into two stages: multi-scale feature extraction and dynamic residual fusion. As shown in Figure 6, firstly, the input features x are extracted by feature grouping and parallel convolutional branching. Specifically, the input features are divided into G subgroups along the channel dimension, each group of features $X_{g}$ is inputted into two parallel convolutional branches, and the features are compressed along the spatial dimension through the global average pooling in the 1 × 1 convolutional branch, which generates the channel-level statistics $z_{g}^{H}$ and $z_{g}^{W}$, which represent the global information in the horizontal and vertical directions, respectively. These two vectors are used to generate channel attention weights through a fully connected layer with a nonlinear activation function to enhance the response of key channels. In the 3 × 3 convolution branch, a local convolution kernel is utilized to capture the spatial details and generate a local feature map $f_{local}$, which preserves the high-frequency detail information. Subsequently, the outputs of the two branches are fused by a cross-space learning strategy, which involves matrix dot product and feature splicing to generate multi-scale features $f_{EMA}\left(x\right)$. This process is represented as

(11) $$\;f_{EMA}\left(x\right)=Concat\left(Softmax\left(\frac{QK^{T}}{\sqrt{d}}\right)V,Conv_{3\times 3}\left(x\right)\right)$$

where *Q*, *K*, and *V* are query, key, and value vectors generated by 1 × 1 convolutional branching, d are feature dimensions, and cross-space interactions achieve global and local feature fusion through the attention mechanism. After obtaining the multi-scale features of $f_{EMA}\left(x\right)$, the WREMA module inputs them with the original input features x into the channel-attention-weighted residual module (CAWR) to realize residual fusion through dynamic weight assignment.

#### 2.2.4. Overall Model Architecture

**Figure 7:**
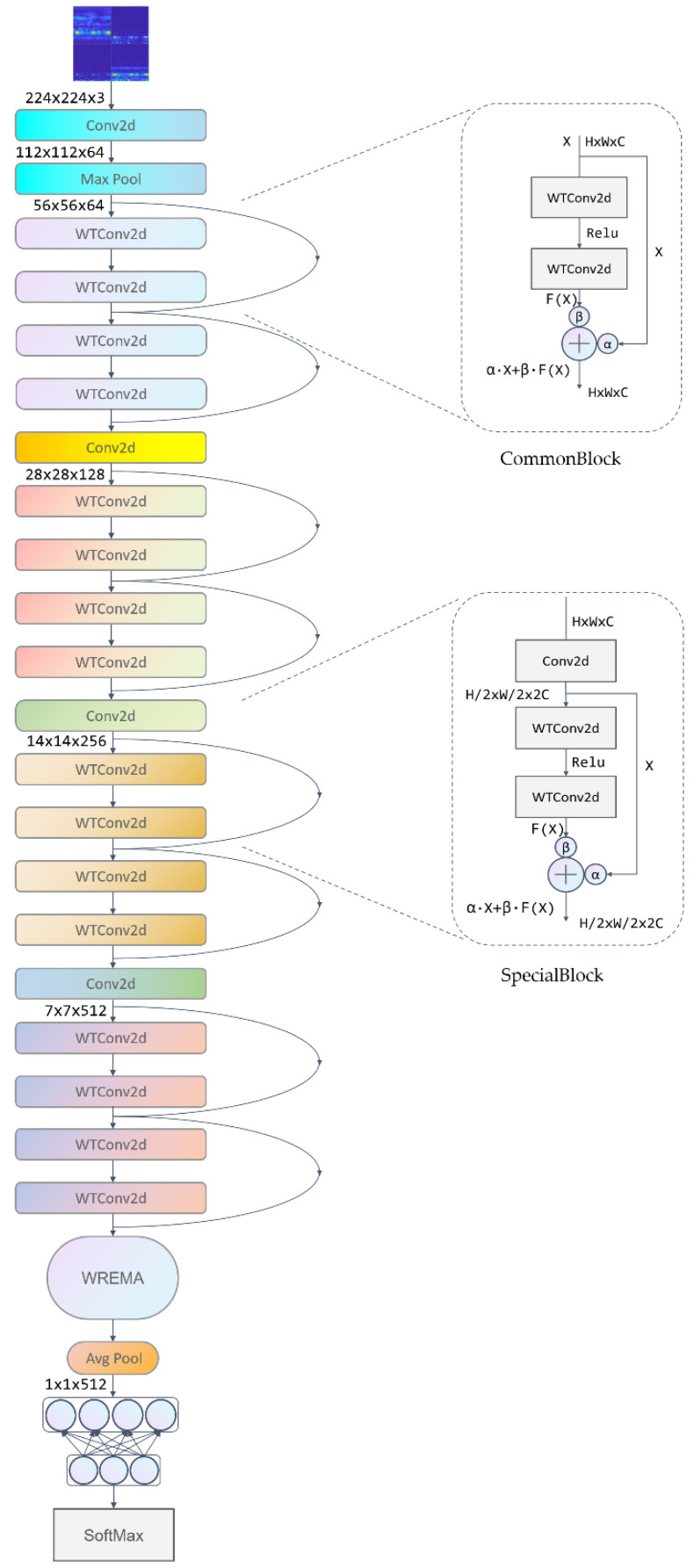
WaveCAResNet architecture.

The bearing fault diagnosis method based on fused time–frequency maps and WaveCAResNet model mainly consists of a wavelet convolution layer, channel-attention-weighted residual (CAWR), a weighted residual block, a weighted residual efficient multi-scale attention (WREMA) with classifiers, and the network architecture is shown in Figure 7.

Firstly, the acquired one-dimensional bearing vibration signals are converted into two-dimensional time–frequency images by time–frequency analysis by connecting *CWT*, *WVD*, *HHT*, and *STFT*, and then the time–frequency maps are fused. The fused time–frequency map is used as the input of WaveCAResNet, and the preliminary feature extraction of the input time–frequency map is performed by a 7 × 7 convolution and maximum pooling, and the preliminary processed feature maps are entered into the backbone network. The model backbone network consists of one CommonBlock weighted residual block, three SpecialBlock weighted residual blocks, and weighted residual efficient multi-scale attention (WREMA).

The CommonBlock weighted residual block is designed for processing features of the same dimensions, and its core architecture consists of a dual-path wavelet convolution (WTConv) mechanism. In this scheme, the first WTConv layer, following batch normalization and ReLU activation, extracts spatial features, while the second WTConv layer keeps the feature dimensions intact. Finally, these features are fused with the original input using a channel-attention-weighted residual (CAWR) mechanism.

SpecialBlock is designed for feature dimension transformation scenarios. It introduces a 1 × 1 convolution with a stride of 2 for downsampling, thereby expanding the input channels to the target dimension. Internally, SpecialBlock employs the same dual wavelet convolution (WTConv) structure as CommonBlock. The downsampled input features are then fused with the convolution outputs via the channel-attention-weighted residual (CAWR) module, ensuring critical fault information is preserved while the feature dimensions are adjusted. Subsequently, feature enhancement is accomplished through the weighted residual efficient multiscale attention (WREMA) module, which extracts temporal correlations from the features using dynamic weighting provided by the CAWR mechanism. An activation function is finally applied to strengthen the nonlinear representation. Once feature extraction is complete, all feature maps are passed sequentially through a global average pooling layer and two fully connected layers before a Softmax layer generates the probability distributions for each fault category, thereby completing the bearing fault classification task.

## 3. Experimental Results and Evaluation

### 3.1. Experimental Settings and Data Sources

In order to verify the superiority, robustness, and generalizability of the proposed model in bearing fault diagnosis, the Case Western Reserve University (CWRU) bearing dataset [29] and the Paderborn University (Paderborn, Germany) bearing dataset [30] were used for experimental validation. The CWRU dataset accurately simulated single-point faults of the inner ring, outer ring, and rolling element on the drive-end bearing by EDM, and the sampling frequency was set to 12 kHz. The CWRU dataset was used to accurately simulate single-point failures on the inner ring, outer ring, and rolling element by EDM, with failure diameters of 0.007 inches, 0.014 inches, and 0.021 inches, and accelerometer vibration signals at the drive-end under a load condition of 0–3 hp, with the sampling frequency set to 12 kHz/48 kHz dual mode, covering different stages of failure progression, from slight to severe. The Paderborn dataset further extends the complexity of failure types to include two types of failure modes, those generated by manual machining (EDM, electro-engraving, drilling) and those generated by natural wear and tear, and involves a variety of failure modes, such as pitting, cracking, surface spalling, etc., with damage sizes spanning from localized micro-cracks (1 mm^2^) to extended spalling (50 mm^2^). The Paderborn dataset, which has been tested by the True The Paderborn data set, was obtained from real bearings through an accelerated life test rig; the experimental conditions are set to cover the speed range of 300–1500 rpm, the radial load span 50–1000 N, and the sampling frequency is 64 kHz high-frequency acquisition in order to retain the transient characteristics of the failure impact.

The vibration signals analyzed in this study were collected from two publicly available datasets using professional industrial-grade equipment: the vibration signals in the CWRU dataset were collected by an accelerometer installed on the drive end of the motor housing and saved after sampling at 12 kHz; the vibration signals in the Paderborn dataset were collected by a piezoelectric accelerometer installed on the bearing housing (Model No. 336C04, PCB Piezotronics, Inc., Depew, NY, USA), digitized, and saved after being sampled at a rate of 64 kHz.

All experiments in this study were conducted on a computer running Windows 10 (64-bit). MATLAB 2023a was employed for the time–frequency analysis of vibration signals, while computational tasks were supported by an NVIDIA RTX 4070 Super GPU and an AMD Ryzen 9 9900X CPU. The proposed WaveCAResNet model was developed using Python 3.9 in conjunction with the PyTorch 2.6.0 deep learning framework. The model architecture parameters are detailed in Table 2.

### 3.2. Assessment of Indicators

To accurately evaluate the functionality and practicality of the model, Accuracy, Precision, Recall, and *F*_1_–*Score* were selected as the core evaluation metrics in this study to comprehensively quantify the model’s classification performance and fault recognition reliability. The formulas are as follows:

(12) $$Accuracy=\;\frac{TP+TN}{TP+TN+FP+FN}$$

(13) $$Precision=\;\frac{TP}{TP+FP}$$

(14) $$Recall=\;\frac{TP}{TP+FN}$$

(15) $$F_{1}\text{-}Score=2\times \;\frac{Precision\times Recall}{Precision+Recall}$$

where *TP* denotes the number of samples where the true category is positive and correctly predicted by the model, *TN* denotes the number of samples where the true category is negative and correctly predicted by the model, *FP* denotes the number of samples where the true category is negative but incorrectly predicted by the model to be positive (false positives), and *FN* denotes the number of samples where the true category is positive but incorrectly predicted by the model to be negative (missed detections).

### 3.3. Experimental Results on the CWRU Dataset

For the CWRU dataset, as detailed in Table 3, a total of 10 fine-grained failure types were considered. This includes one normal condition and three fault conditions—inner ring failure, outer ring failure, and ball failures—each with three damage diameters (0.007 inch, 0.014 inch, and 0.021 inch). The data were collected at the drive end under a 0-load condition with a sampling frequency of 12 kHz.

#### 3.3.1. Comparative Experiments

**Figure 8:**
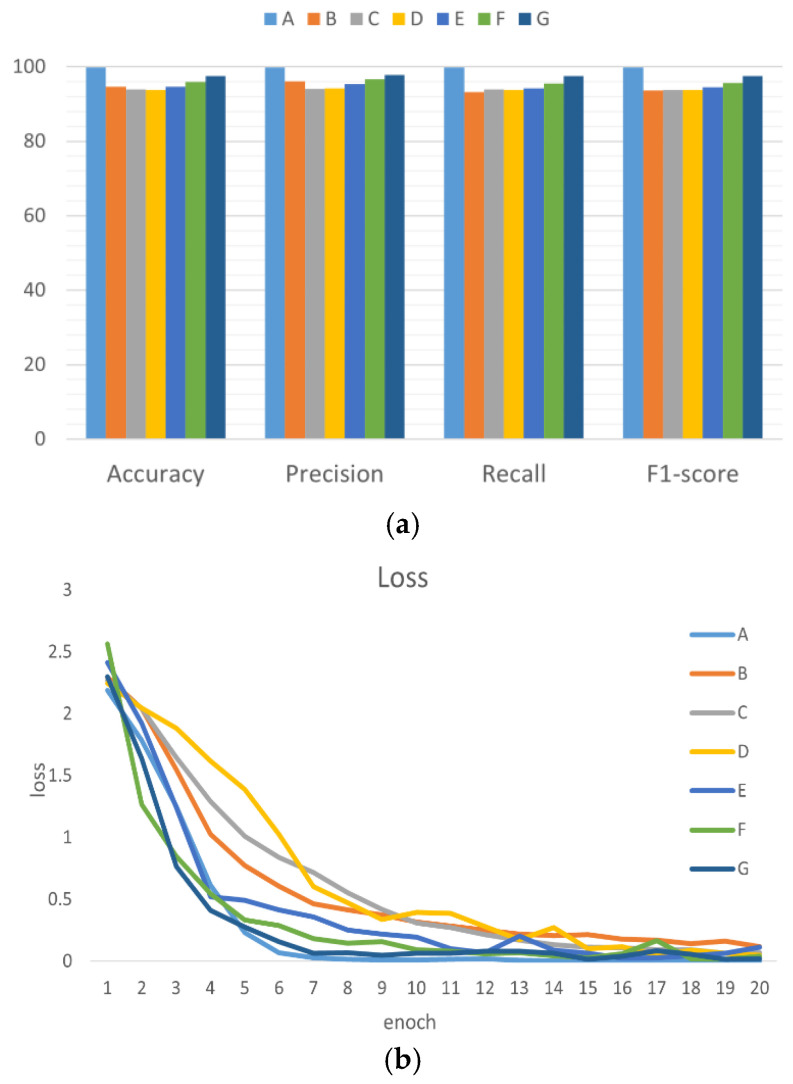
Results of comparative experiments: (**a**) comparison of model performance (A–G at the top of the image represent the model groups, the horizontal axis represents the evaluation criteria used in this study, and the vertical axis represents the corresponding values of the evaluation criteria) and (**b**) comparison of the loss of each model.

To validate the performance of the proposed WaveCAResNet model for bearing fault diagnosis, we conducted comparative experiments with six mainstream deep learning models. The selected models include representative convolutional neural networks such as AlexNet [31], MobileNet-V2 [32], and EfficientNet-b0 [33], as well as Vision Transformer-based architectures and their variants, namely ViT-Base [34], ConvNeXt-T [35], and SwinT [36]. Among these, AlexNet, MobileNet-V2, and EfficientNet-b0 showcase a lightweight and classical CNN design, while ViT-Base, ConvNeXt-T, and SwinT reflect the evolutionary trends of the Transformer architecture. Table 4 summarizes these models, and Figure 8 illustrates the comparison results on the CWRU dataset.

As shown in Figure 8, the proposed WaveCAResNet model significantly outperforms all compared models, achieving an average accuracy of 99.86%. This represents an improvement of 2.32 percentage points over the next best model, SwinT, 3.97 percentage points over ConvNeXt-T, and 5.88 percentage points over the lightweight MobileNet-V2. Although ViT-Base effectively captures global features, its performance is limited by high computational complexity and challenges in modeling local details, resulting in an accuracy of 94.64%. The results indicate that WaveCAResNet precisely models the time–frequency characteristics of bearing faults by integrating the multi-scale frequency domain feature extraction capability of wavelet convolution (WTConv), the dynamic fusion mechanism of channel attention-weighted residuals (CAWR), and the spatial attention enhancement provided by WREMA. Additionally, its computational efficiency of only 0.2 GFLOPs substantially outperforms ViT-Base (16.86 GFLOPs) and SwinT (4.37 GFLOPs), demonstrating the model’s feasibility for deployment on industrial edge devices.

#### 3.3.2. Visualization of Model Results

**Figure 9:**
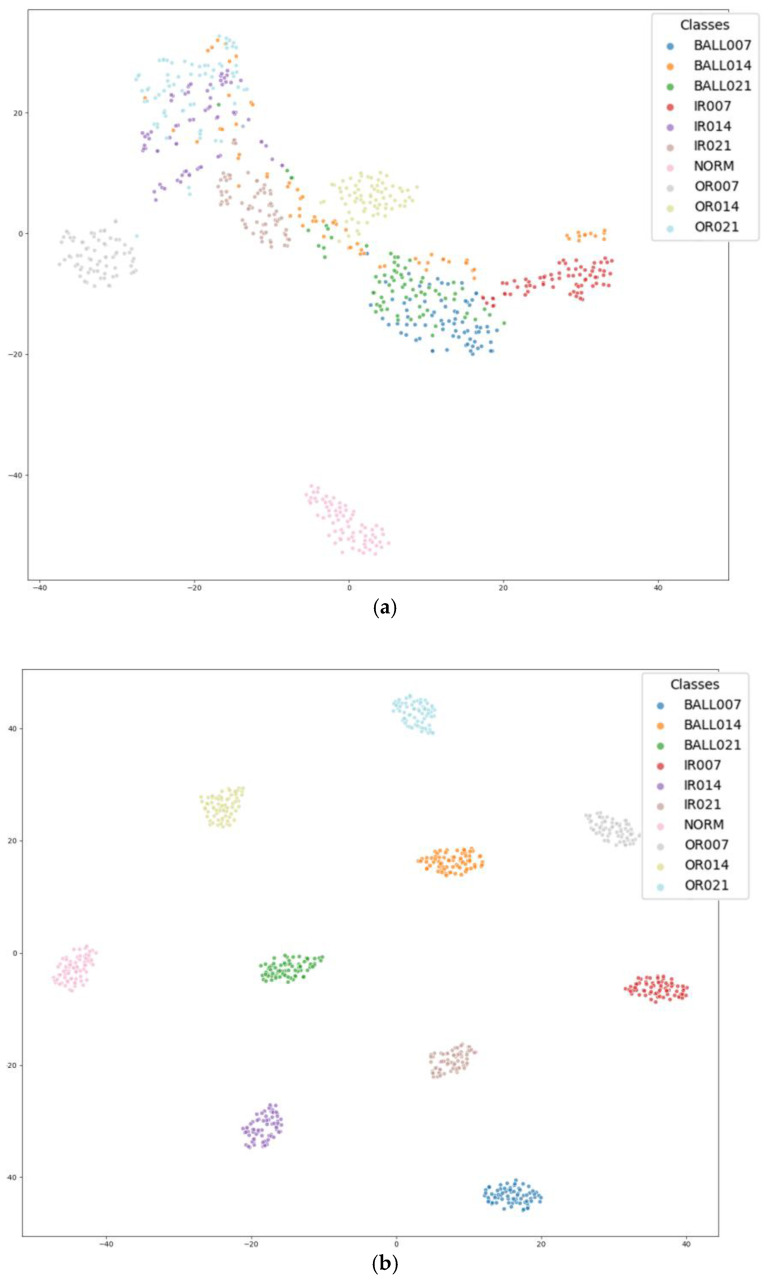
Visualization of results: (**a**) initial distribution and (**b**) output distribution.

In fault diagnosis tasks, feature visualization serves as an essential method to validate the learning ability of models. Because the abstract features extracted in high-dimensional space are often difficult to interpret directly, dimensionality reduction techniques are used to map these features into two- or three-dimensional spaces. This transformation allows for a visual assessment of the separability of different fault categories and the overall reasonableness of the feature distribution. In this study, we employ the t-Distributed Stochastic Neighbor Embedding (t-SNE) algorithm for feature visualization. This algorithm nonlinearly maps high-dimensional features to a low-dimensional space while preserving local structures, resulting in clearly separated clusters for different fault types in the two-dimensional space. Figure 9 presents the resulting feature distribution obtained from the dataset.

The dynamic evolution of the feature representation during model training is clearly demonstrated in the feature visualization results presented in Figure 9. Figure 9a illustrates the high-dimensional feature distribution in the untrained initial state, where sample points from different fault categories exhibit significant spatial overlap and intermingling. This indicates that, in the original feature space, the discriminative information of the fault modes has not been effectively extracted. As the model training progresses and optimization continues, Figure 9b reveals a notable structural transformation in the feature space: similar fault samples begin to form high-density clusters in the low-dimensional projection, while distinct fault categories develop clear decision boundaries. The 10 fault categories were clearly distinguished, and the cluster boundaries were very clear. This evolution—transitioning from a ‘chaotic distribution’ to a state characterized by ‘tight intra-class cohesion and clear inter-class separation’—validates the model’s robust ability to distinguish between faulty and fault-free data.

### 3.4. Experimental Results on the Paderborn Dataset

For the Paderborn dataset, the focus is on complex failure modes arising from manual machining defects, including crack damage in EDM, spalling damage in drilling, and pitting damage in electric engraving. As detailed in Table 5, the bearing states comprise normal, inner ring damage, and outer ring damage, thereby fully simulating the multi-source coupled faults commonly encountered in industrial settings. Consequently, a classification system with 13 fine-grained fault types is established.

#### 3.4.1. Comparative Experiments of Time-Frequency Analysis Methods

To validate the superiority of the fused time–frequency map method proposed in this study for bearing fault diagnosis tasks, we conducted comparative experiments evaluating the classification performance on the WaveCAResNet model. The evaluated methods included

- Four single time–frequency representation analysis methods (*CWT*, *STFT*, *HHT*, *WVD*);
- Channel-overlay time–frequency map method (generated by superimposing time–frequency map derived from three analytical techniques—*CWT*, *STFT*, and *WVD*—onto corresponding RGB channels);
- The spatially fused time–frequency map method is introduced in this study.
- The experimental groupings are detailed in Table 6.

**Figure 10:**
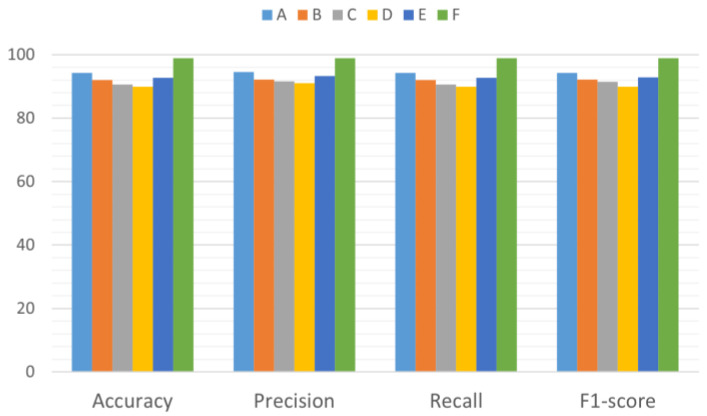
Comparison of experimental results (A–F at the top of the image represent the groups of methods used, the horizontal axis represents the evaluation criteria used in this study, and the vertical axis represents the corresponding values of the evaluation criteria).

The comparison results are shown in Figure 10. The fusion time–frequency map method proposed in this study significantly outperforms other methods with an accuracy rate of 98.89% (CWT: 94.27%; STFT: 92.06%; HHT: 90.55%; WVD: 89.95%; Overlay: 92.76%). Additionally, its precision rate, recall rate, and F1 score also reached 98.92%, 98.89%, and 98.90, respectively.

**Table 6:** Experimental grouping.

Time–Frequency Analysis Methods	Groups
CWT	A
STFT	B
HHT	C
WVD	D
Channel-overlay time–frequency map method	E
fused time–frequency map method	F

**Figure 11:**
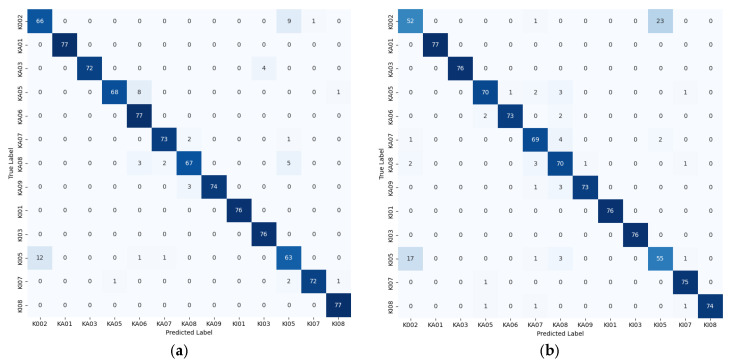

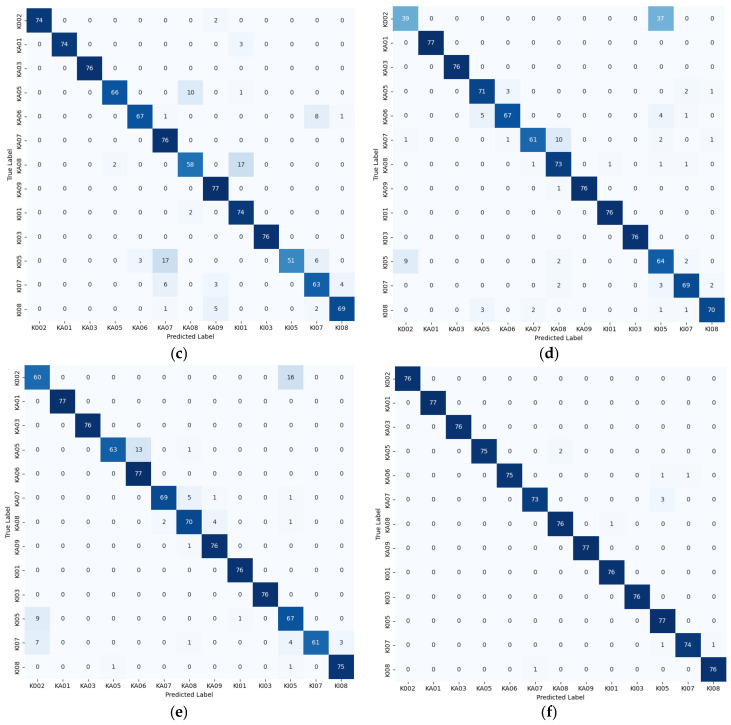
Confusion matrix of diagnostic results for each method: (**a**) CWT method; (**b**) STFT method; (**c**) HHT method; (**d**) WVD method; (**e**) channel-overlay time–frequency map method; and (**f**) fused time–frequency map method.

The confusion matrix is an intuitive and effective tool for evaluating classification models. It presents, in tabular form, the relationship between the predicted labels and the actual labels, thereby reflecting the diagnostic performance of the model for each fault category. In this study, the model’s discriminative capabilities are visualized using the confusion matrix, as shown in Figure 11.

The confusion matrices shown in Figure 11 illustrate the classification performance of various fault diagnosis methods. In these matrices, each row corresponds to the true label, and each column represents the predicted label. The diagonal values indicate the number of samples correctly classified by the model, thereby reflecting its overall accuracy. By examining these matrices, we can visually compare the performance differences of the various methods across different fault types.

Specifically, the CWT matrix generally performs well, though some categories (e.g., k002, KA05, KA08, KI05) are misclassified. The *STFT* matrix achieves high accuracy overall, yet K002 and KI05 still show some errors. In contrast, the *HHT* matrix exhibits poorer performance with numerous misclassifications, and the *WVD* matrix records a notably higher error rate for K002. Furthermore, the channel-overlaid time–frequency map method misclassifies samples such as K002, KA05, and KI07, while the fused time–frequency map method nearly perfectly classifies all fault categories.

By analyzing the results of the confusion matrix shown in Figure 11, the classification performance of various time–frequency analysis methods for bearing fault diagnosis becomes apparent. The confusion matrix highlights the significant superiority of the fusion time–frequency map (fused time–frequency map method) in diagnosing bearing faults. Specifically, compared to single time–frequency analysis methods (e.g., *CWT*, *STFT*, *HHT*, *WVD*) and the channel-overlay time–frequency map method, the proposed fused time–frequency map method achieves a remarkably high classification accuracy of 98.89%, significantly outperforming the others, which reach accuracies of up to 94.27%. Moreover, the confusion matrix demonstrates that the fused time–frequency map method markedly improves prediction accuracy across multiple fault categories while considerably reducing misclassifications, particularly in the recognition of critical fault types. These findings underscore the effectiveness and adaptability of the proposed method in addressing complex fault patterns.

#### 3.4.2. Ablation Experiments

**Figure 12:**
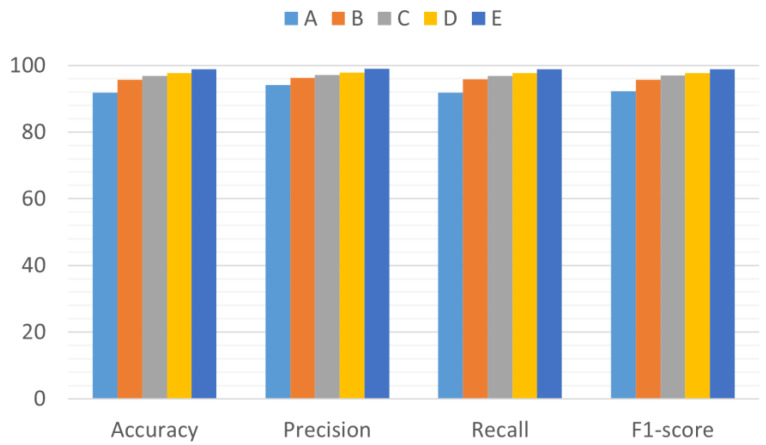
Results of ablation experiments (A–E at the top of the image represent the model groups, the horizontal axis represents the evaluation criteria used in this study, and the vertical axis represents the corresponding values of the evaluation criteria).

To verify the effectiveness of the proposed WaveCAResNet model, ablation experiments were conducted using the Paderborn dataset, as summarized in Table 7. Specifically, Experiment A employed the standard ResNet-18, Experiment B introduced wavelet convolution to create WTResNet, Experiment C incorporated an Exponential Moving Average (EMA) mechanism into WTResNet to form WT-EMA-ResNet, and Experiment D further integrated a weighted residual EMA (WREMA) mechanism, resulting in WT-WREMA-ResNet. As illustrated in Figure 12, the original ResNet-18 achieved an accuracy of only 91.86% due to traditional convolution kernels in capturing time–frequency localized details. By introducing wavelet convolution (WTConv), the model significantly enhanced its transient feature extraction capability via multi-scale band decomposition, improving accuracy to 95.76%. The addition of EMA in WT-EMA-ResNet further leveraged multi-scale attention to focus on fault-sensitive regions, increasing accuracy to 96.88%. Integrating WREMA optimized the EMA feature fusion path through a residual weighting mechanism, further boosting accuracy to 97.69%. Finally, the WaveCAResNet model combined the weighted residual strategy with the CAWR mechanism in its residual blocks to achieve an accuracy of 98.89%. Overall, the incremental contributions of the wavelet convolution, WREMA, and the CAWR mechanism yielded accuracy gains of 3.9%, 1.93%, and 1.2%, respectively, thereby confirming the effectiveness of the proposed approach.

#### 3.4.3. Model Performance in Noisy Environments

In practical engineering applications, bearings operate in complex conditions with variable working environments, where noise in the signals is inevitable. To mimic bearing failures under diverse environmental conditions and to comprehensively assess the performance of the WaveCAResNet model alongside other compared models under different noise levels, this study introduces white noise at various signal-to-noise ratio (*SNR*) levels during experiments. The SNR is defined by the following formula:

(16) $$SNR_{dB}=10\log_{10}\left(P_{signal}/P_{noise}\right)\;$$

where $P_{\mathrm{signal}}$ denotes the power of the signal and $P_{\mathrm{noise}}$ denotes the power of the noise.

**Figure 13:**
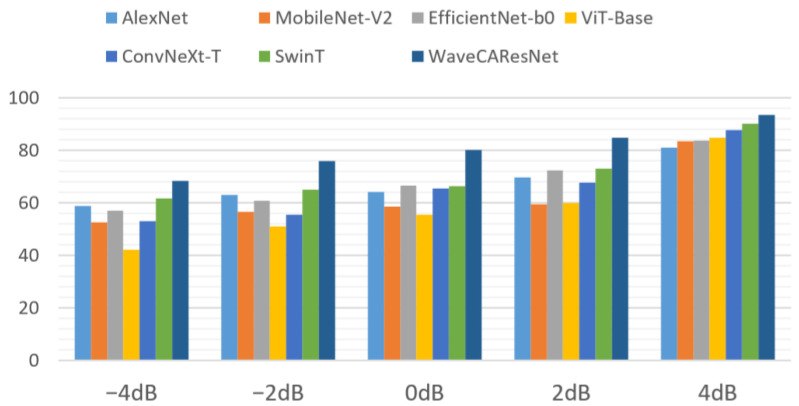
Accuracy of different models at each signal-to-noise ratio (the labels at the top of the image represent the model groups, the horizontal axis represents the signal-to-noise ratio (SNR), and the vertical axis represents the accuracy of each model at each signal-to-noise ratio (SNR)).

Experiments were conducted by adding −4 dB, −2 dB, 0 dB, 2 dB, and 4 dB of white noise in the dataset to simulate the actual noise scenarios in the production environment. The accuracy of different models at each signal-to-noise ratio is shown in Figure 13.

As shown in Figure 13, model performance comparisons under noisy conditions reveal notable differences in noise robustness. When the signal-to-noise ratio (SNR) decreases from 4 dB to −4 dB, traditional convolutional networks such as AlexNet and the lightweight MobileNet-V2 exhibit clear performance degradation under low-SNR conditions due to their fixed convolutional kernels’ inability to isolate high-frequency fault features from strong noise. Although EfficientNet-B0 achieves improved efficiency in noise-free scenarios through structural optimization, its shallow feature extraction capabilities become limited under noisy disturbances. Within Transformer-based architectures, ViT-Base experiences the most significant performance drop because its global attention mechanism is highly sensitive to noise and tends to disperse attention weights to non-critical regions at low SNRs. While the Swin Transformer partly alleviates this issue through hierarchical windowed attention, it still struggles with ambiguous feature characterization when the signal is overwhelmed by noise. Similarly, the improved convolutional network ConvNext-T approaches the performance of Swin Transformer at high SNR levels, yet its fault discrimination ability is progressively compromised as noise increases. In contrast, the proposed WaveCAResNet model employs a multi-mechanism synergistic design, integrating a wavelet convolutional layer (WTConv), a CAWR mechanism, and the WREMA module to achieve significantly stronger noise robustness and generalization capabilities.

#### 3.4.4. Verification of Frequency Response of Fault Characteristics

**Figure 14:**
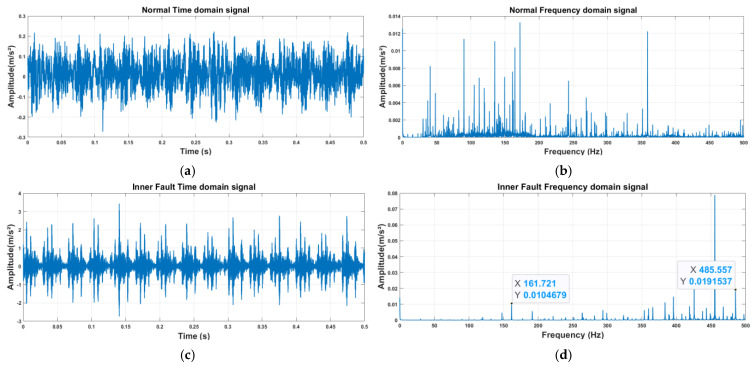

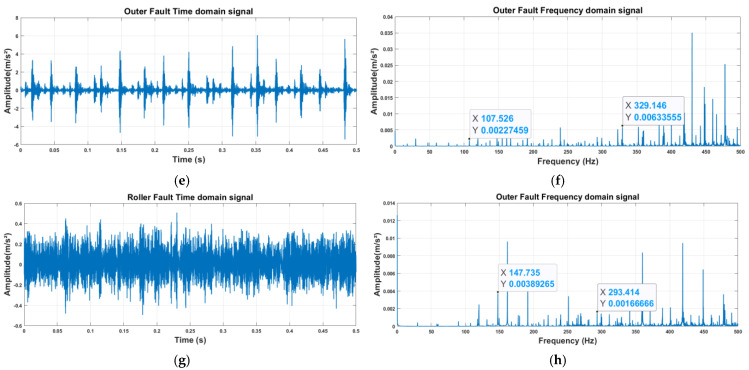
Time-domain and frequency-domain signals of bearing vibrations under different faults: (**a**) NOT; (**b**) NOF; (**c**) IFT; (**d**) IFF; (**e**) OFT; (**f**) OFF; (**g**) RFT; and (**h**) RFF.

Although the confusion matrix and clustering results confirm the model’s high diagnostic accuracy (99.86%), understanding the physical causes of fault characteristics is vital for industrial applications. This study performed envelope analysis on vibration signals from healthy and faulty bearings by comparing the spectra of healthy bearings, inner ring faults, outer ring faults, and rolling element faults from the CWRU dataset. Based on the provided bearing parameters (rolling element diameter: 7.94 mm, pitch diameter: 39.04 mm, contact angle: 0°), the fault frequencies were calculated as follows: inner ring fault (BPFI) at 162.18 Hz, outer ring fault (BPFO) at 107.36 Hz, and rolling element fault (BSF) at 141.66 Hz. As shown in Figure 14, since low-frequency signals are rich in system state information, the analysis focused on the first 0.5 s and the first 500 Hz, following the settings referenced in [37].

The spectral distribution of healthy bearings is relatively uniform. Inner ring faults exhibit a distinct peak at 162.721 Hz, while outer ring faults show a significant increase in energy at 107.526 Hz and its second harmonic at 214.72 Hz. Rolling element faults reach a peak at 147.727 Hz. All three fault types are highly consistent with theoretical fault characteristic frequencies, and distinct peaks are also observed at the corresponding harmonic frequencies. The frequency response deviations between healthy bearings, inner ring faults, outer ring faults, and rolling element faults effectively explain the differences in fault mechanisms from a physical perspective.

## 4. Conclusions

This study proposes a novel framework, WaveCAResNet, which integrates multi-source time–frequency analysis with lightweight deep learning to address issues including signal non-stationarity, noise interference, and limited feature characterization that often plague conventional fault diagnosis methods for rolling bearings. By harnessing the complementary strengths of multiple time–frequency transformations, the framework effectively overcomes the shortcomings of a single time–frequency analysis approach and facilitates a comprehensive capture of complex fault characteristics. In terms of model design, the wavelet convolutional layer (WTConv) extends the extraction capability for both local and global features via multi-scale decomposition; the channel-attention-weighted residual module enhances cross-layer feature fusion through dynamic weight allocation; and the weighted residual efficient multi-scale attention further strengthens the synergistic modeling of temporal dependencies and spatial features. Experimental results demonstrate that the proposed method significantly outperforms traditional models across multiple benchmark datasets and exhibits robust performance in noisy environments. Ablation studies further validate the synergistic effect of each module on performance improvements, confirming the effectiveness of the overall design. This study not only provides a high-precision and low-complexity solution for bearing fault diagnosis but also offers a design concept based on multimodal feature fusion and lightweight network architecture that can be extended to other intelligent operation and maintenance applications for rotating machinery. Future research will explore the generalizability of various time–frequency combinations and further optimize model deployment efficiency to satisfy industrial real-time requirements.

## Figures and Tables

**Table 1:** Motor and bearing parameters.

Parameter Category	CWRU Dataset	Paderborn Dataset
Motor parameters	Motor type: 1.5 kW (2HP) induction motor	Drive motor: Haning synchronous motor (425 W, 3000 RPM, 1.35 Nm) Motor type: 1.5 kW (2HP) induction motor
	Speed range: 1730–1797 RPM	Load motor: Siemens synchronous servo motor (1.7 kW, 3000 RPM, 6 Nm)
Bearing parameters	Model: SKF 6205-2RS	Model: Ball Bearing 6203
	Inner diameter: 25 mm	Inner diameter: 30 mm
	outer diameter: 52 mm	outer diameter: 55 mm
	Number of rolling elements: 9	Number of rolling elements: 12

**Table 2:** Hyperparameters of models.

Block	Layers	Training Settings	Hyperparameterization	Settings	Quantities
-	Conv2d	Epoch = 20 Batch Size = 32 Learning Rate = 0.001 Loss Function = CrossEntropyLoss Optimizer = Adam	kernel	7 × 7	1
			Stride	2	
			Padding	3	
			channels	64	
CommonBlock	WTConv2d		kernel	3 × 3	[2, 1, 1, 1]
			Stride	1	
			number	2	
SpecialBlock	Conv2d		kernel	1 × 1	[0, 1, 1, 1]
			Stride	2	
			channels	[128, 256, 512]	
			number	1	
	WTConv2d		kernel	3 × 3	
			Stride	1	
			number	2	
CAWR	Linear		dimensionality reduction ratio	16	9
WREMA	GroupNorm		Number of groups	8	1

**Table 3:** CWRU dataset segmentation.

Bearing Condition	Failure Size/Inch	Labels
normal	-	NORM
inner-ring failures	0.007	IN007
	0.014	IR014
	0.021	IR021
outer-ring failures	0.007	OR007
	0.014	OR014
	0.021	OR021
ball failures	0.007	BALL007
	0.014	BALL014
	0.021	BALL021

**Table 4:** Comparison of experimental setups.

Model	Size (MB)	Quantity of Participants (M)	FLOPs (G)	Groups
WaveCAResNet	4.7	1.07	0.2	A
AlexNet	55.7	14.6	0.28	B
MobileNet-V2	8.7	2.24	0.33	C
EfficientNet-b0	15.6	4.02	0.41	D
ViT-Base	327.4	85.81	16.86	E
ConvNeXt-T	104.2	27.83	4.45	F
SwinT	105.3	27.53	4.37	G

**Table 5:** Paderborn dataset segmentation information.

Bearing Condition	Extent of Damage (Level)	Damage Method	Labels
normal	-	-	K002
outer-ring failures	1	EDM	KA01
	2	electric engraver	KA03
	1	electric engraver	KA05
	2	electric engraver	KA06
	1	drilling	KA07
	2	drilling	KA08
	2	drilling	KA09
inner-ring failures	1	EDM	KI01
	1	electric engraver	KI03
	1	electric engraver	KI05
	2	electric engraver	KI07
	2	electric engraver	KI08

**Table 7:** Experimental group.

Model	Groups
ResNet-18	A
WTResNet	B
WT-EMA-ResNet	C
WT-WREMA-ResNet	D
WaveCAResNet	E

## Data Availability

The CWRU dataset is available at https://engineering.case.edu/bearingdatacenter (accessed on 24 January 2025), and The Paderborn dataset is available at https://groups.uni-paderborn.de/kat/BearingDataCenter (accessed on 28 January 2025).

## Abbreviations

The following abbreviations are used in this manuscript:

CWT	continuous wavelet transform
STFT	Short Time Fourier Transform
HHT	Hilbert-Huang Transform
WVD	Wigner-Ville distribution
CNN	convolutional neural network
ReLU	rectified linear unit
